# Gastro-colo-bronchial fistula after laparoscopic sleeve gastrectomy; case report

**DOI:** 10.1016/j.amsu.2020.05.033

**Published:** 2020-05-30

**Authors:** Ayad Ahmad Mohammed, Sardar Hassan Arif

**Affiliations:** Department of Surgery, College of Medicine, University of Duhok, Kurdistan Region, Iraq

**Keywords:** Sleeve gastrectomy, Leak, Gastrobronchial fistula, Bariatric surgery, Endoscopic stent, Colonic fistula

## Abstract

Obesity and its related comorbidities is a major health problem worldwide. Sleeve gastrectomy is regarded to be one of the most effective bariatric surgeries with a relatively low risks of complications. Gastrobronchial fistula is an extremely rare and a serious complication after bariatric surgeries, it is associated with major morbidity.

A 48-year-old obese lady with a BMI of 40 had underwent laparoscopic sleeve gastrectomy 7 years ago, she developed leak at the 10th postoperative day which was treated with drainage. After 4 years she presented with left subphrenic abscess which was treated with drainage, splenectomy and endoscopic stent. After one year she had repeated chest infections and was coughing-up recently ingested food items. CT-scan showed left subphrenic collection with abnormal fistulous tract between the bronchial tree and the subphrenic cavity. Left thoracotomy was performed, a complex fistula was found between the remnant parts of the gastric fundus, transverse colon and lung. Resection of the fistula was performed, the stomach and colon were closed in 2 layers, resection of the affected segment of lung was performed and the diaphragm was sutured. The BMI was 19 at the last admission.

Gastro-colo-bronchial fistula is unreported after sleeve gastrectomy and the management is challenging. Surgeons may follow the same principles of management as in cases of gastrobronchial fistula, but we suggest earlier surgical intervention with the administration of broad spectrum antibiotics. Nutritional deficiencies must be corrected, and such patients must be treated with multidisciplinary team, with an extended duration of follow-up.

## Highlights

• Sleeve gastrectomy is one of the most effective bariatric surgeries with a relatively low complication rates.
• Gastro-colo-bronchial fistula is unreported after sleeve gastrectomy and the management is challenging.
• Multidisciplinary team is required for the management of such patients.

## 1 Introduction

Obesity and its related comorbidities is a major health problem worldwide. The number of obese people is increasing due to unhealthy eating habits and lack of exercise [1].

Currently there many surgical and endoscopic management options for the treatment of obesity, such as intra-gastric balloon placement, sleeve gastrectomy and bypass surgeries, some act by restricting the food intake, others by decreasing the absorption of the ingested food, or by the combined restrictive and the malabsorptive mechanisms [2].

Complications after sleeve gastrectomy are estimated to occur in up to 5% of patients, leak after sleeve gastrectomy is the most serious complication and is estimated to occur in 0.5–1% of patients, it is generally classified to be either subclinical (type I) or clinical (type II) leaks depending on the radiological appearance and the clinical condition of the patient. In subclinical leaks the patients is clinically stable and is limited to the site of surgery, while in the clinical leaks there is free intra-peritoneal leak and the patient's conditions is clinically unstable. Some authors may classify leaks to be either acute if it occurs in less than 3 days or chronic if it occurs after 8 days. The risk of gastric fistula after sleeve gastrectomy is estimated in some literature to be around 0.9%–2.6% after the first surgery, and up to 8% after the second surgery [1,3].

Patients presented with recurrent chest infections which are difficult to treat, in addition to signs of leak such as epigastric pain, fever, vomiting, sometimes in rare occasions patients may present with expectoration of food particles such as the presentation in the presented case. When the leak is free intra-peritoneal, patients presented with generalized abdominal pain, and signs of septic shock. Most patients have leak prior to the development of fistula [3,4].

To the best of our knowledge this is the first reported case that involve the stomach, the colon, and the bronchial tree, although some cases are reported that involve fistula between the stomach and the bronchial tree.

The work in this case report has been reported in line with the SCARE 2018 criteria [5].

### 1.1 Patient information

**Clinical findings:** A 48-year-old obese lady with a BMI of 40, she decided to consult the surgical unit for weight reduction surgery after failure of other means for reducing weight such as diet and exercise. The patient underwent laparoscopic sleeve gastrectomy 7 years ago. After surgery she developed leak from the staple line at the 10th postoperative day and she was presented with fever and left sub-phrenic abscess which was drained at that time with drainage and drain placement. The patient did well after that and she was discharged home after that and she attended regular follow up visits for 1 year.

The patient is a known case of diabetes mellitus for the last 5 years which was poorly controlled with diet and oral hypoglycemic drugs.

After 4 years she presented with left hypochondrial pain and fever. CT-scan showed evidence of recurrent left subphrenic abscess with left side pleural effusion. She was diagnosed as leak from the stomach with infection, endoscopic gastric stent was placed. Laparotomy was performed and the abscess was drained, during surgery incidental splenic injury occurred and splenectomy was performed, tube drain was placed for 5 days, the patient received antibiotics for 10 days.

After one year she had frequent presentations for repeated attacks of chest infection and pleural effusions, repeated aspirations were performed for the effusion and she was admitted 2 times to the hospital and received parenteral antibiotics.

The patient developed cough after meals with coughing-up recently ingested food items. She was admitted to the hospital, during examination, the pulse rate was 105 beats/minute, the blood pressure was 100/55 mmHg, and the temperature was 38.3° of Celsius. The patient was pale and the BMI was 19.

**Diagnostic assessment**: The WBC count were 12000/cmm, the albumin was 2.8 g/L, and the hemoglobin was 8.9 g/dl.

**Fig. 1:**
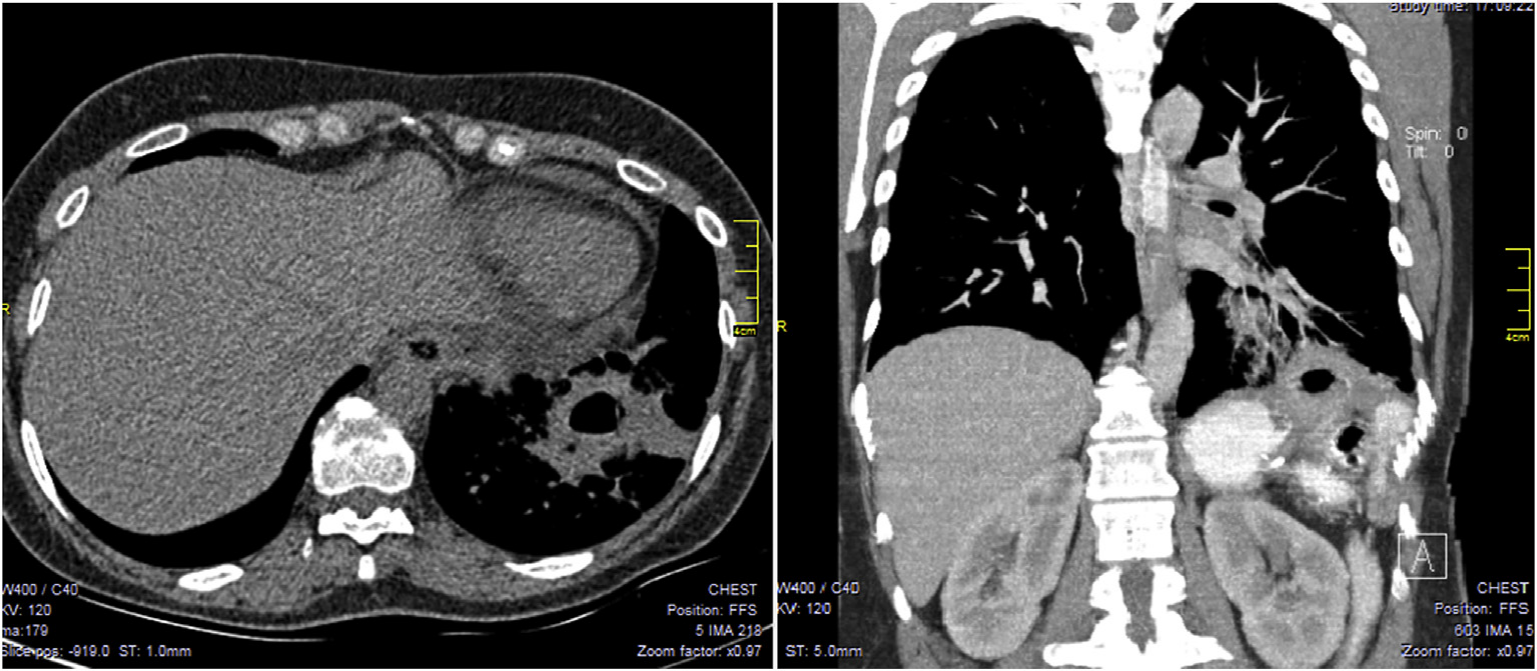
CT-scan of the chest and the upper abdomen showing an evidence of collection in the left sub-diaphragmatic region and consolidation of the left lung zone.

CT-scan showed an evidence of subphrenic collection with suspicion of abnormal fistulous tract between the bronchial tree and the subphrenic cavity. Fig. 1.

**Fig. 2:**
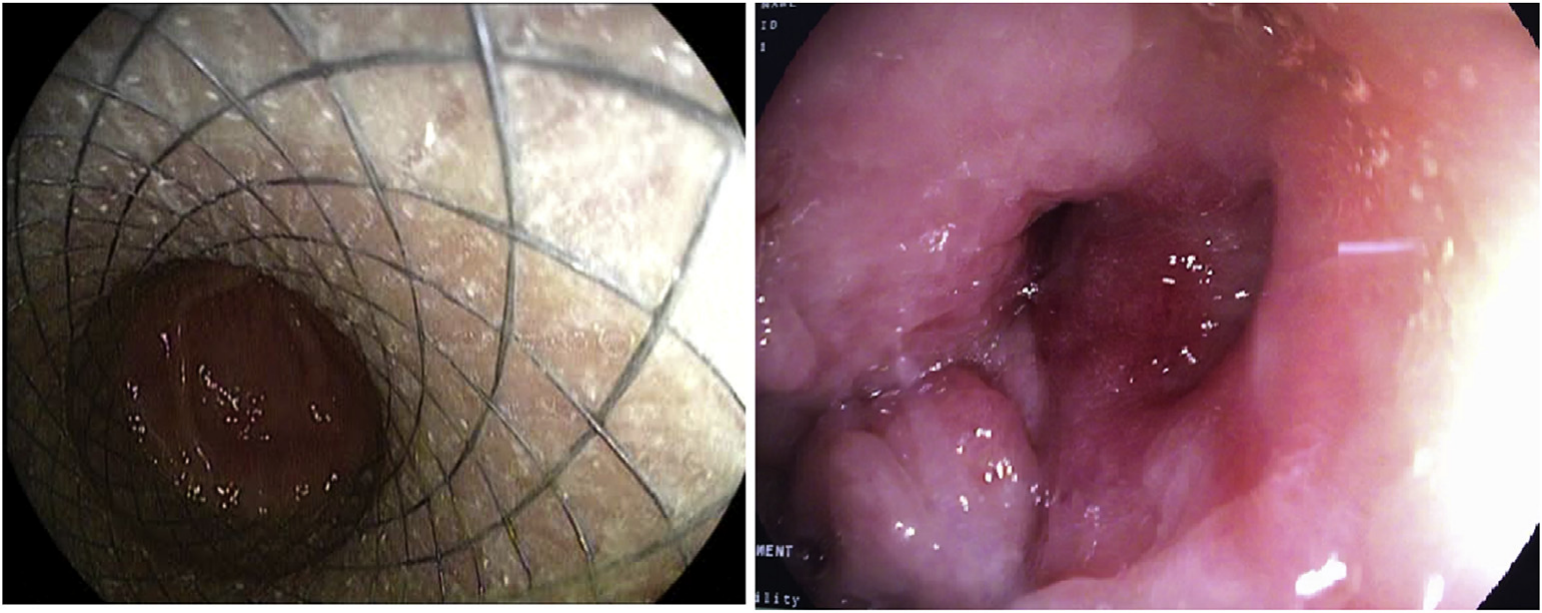
An endoscopic view showing the stent inside the gastric remnant, the site of the leak is evident as an abnormal opening.

Endoscopy showed the stent inside with abnormal opening with surrounding edema suggesting the site of leak. Fig. 2.

The patient received 2 units of compatible blood and albumin, with parenteral antibiotics.

**Fig. 3:**
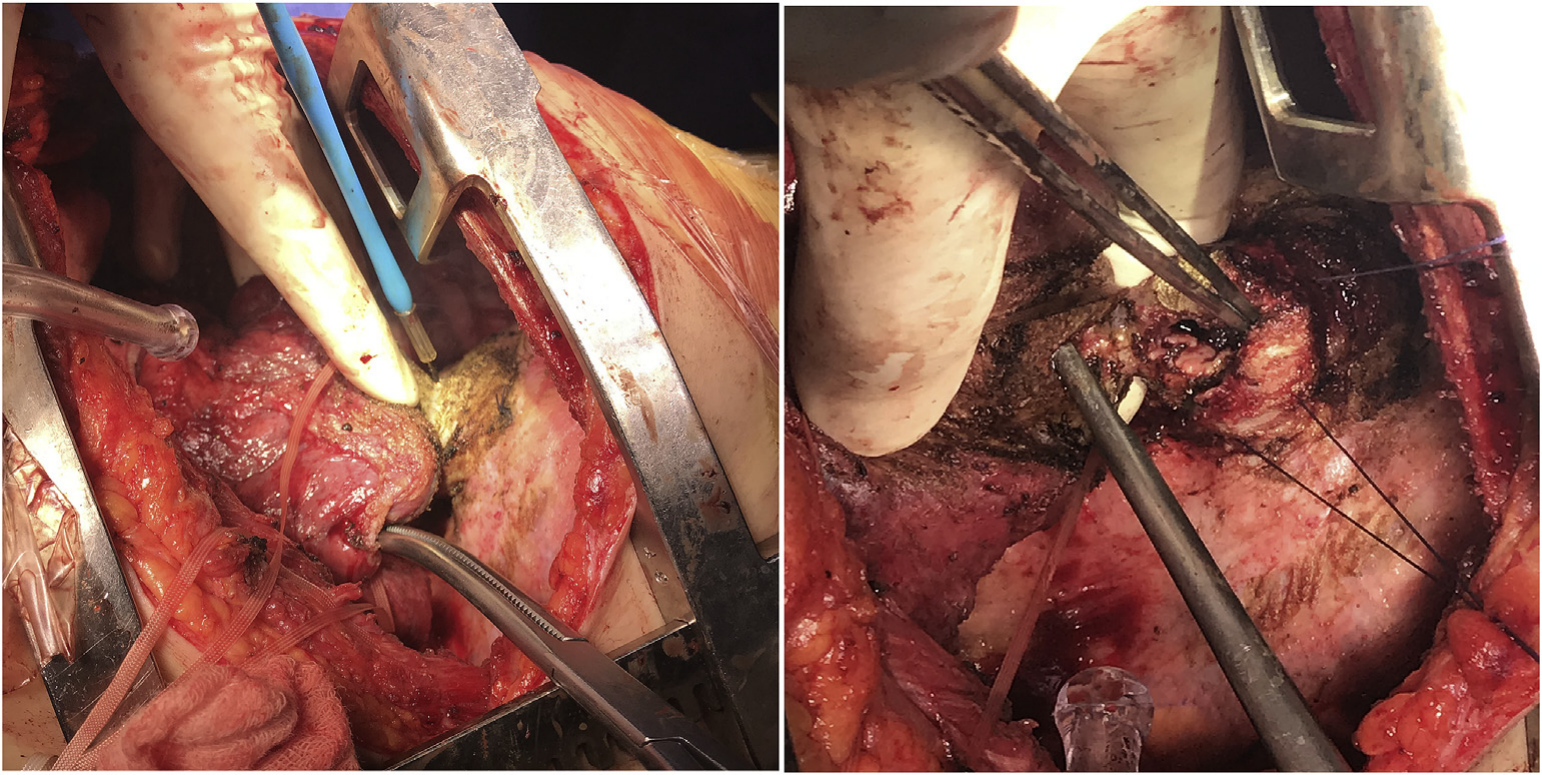
An intraoperative picture showing the abnormal fistulous tract connecting the remnant of the gastric fundus, the colon and the lower lung lobe.

**Therapeutic Intervention**: Decision was done for left thoracotomy, during operation an abnormal fistulous tract was found between the remnant parts of the gastric fundus, the transverse colon and left lower lobe of the lung. Fig. 3.

The diaphragm was opened and refreshment of the edges of the diaphragm was performed, resection of the fistulous tract was performed and the stomach and the colonic walls were closed in 2 layers with a slowly absorbable suture material. Resection of the affected segment of left lower lobe of the lung was performed and the diaphragm was sutured.

**Fig. 4:**
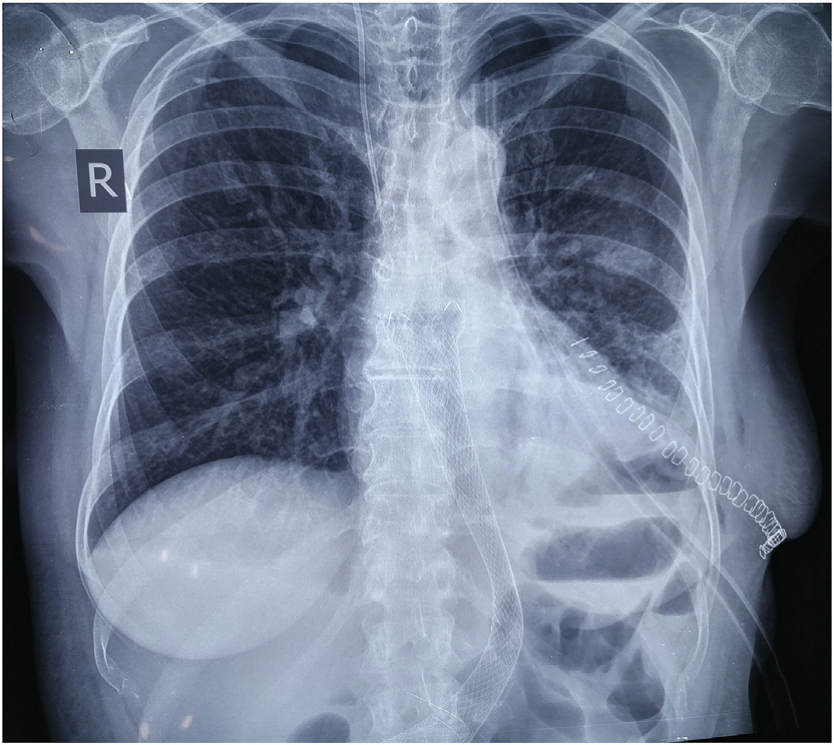
Post-operative CXR showing full lung expansion, the stent in its place, the postoperative chest X-ray and the metallic skin clips.

Post-operative chest X-ray showed full lung expansion with no evidence of collection. Fig. 4.

**Follow-up and outcomes**:Left chest drain was put and she was admitted to the intensive care unit for 3 days, the chest drain was removed at day 4, oral intake was started at day 3, and she was discharged at day 8 after surgery. Follow up was done for 6 months after surgery with no complications, she was advised to attend regular follow-up visits and with supervision of nutritionist and rehabilitation therapist.

## 2 Discussion

Sleeve gastrectomy is a relatively safe procedure and most patients are satisfied with the results of surgery and weight reduction [1].

Gastrobronchial fistula is an extremely rare and a serious complication after bariatric surgeries, it is associated with major morbidity. The incidence is higher after bypass surgery [6].

The general condition of the patients should be stabilized with correction of anemia and nutritional deficiencies before any major surgical intervention to maximize the success rate after surgery, a multidisciplinary medical team is mandatory for effective management [7].

In a review of literature, cases with gastrobronchial fistula were treated with a variety of treatment options as currently there is no a standard form of management. Patients who develop lung abscess are treated with thoracotomy and antibiotics, other patients with intra-abdominal collections are treated with percutaneous drainage when the collection is minor and placement of stent, patients with major intra-abdominal collections and who are septic are usually treated with abdominal reoperation and placement of nutritional access. Patients with stricture are treated with balloon dilatation and stent placement [6].

In selected patients the endoscopic placement of fibrin glue may be tried with variable degree of success, this technique usually required multiple sessions [6].

Some authors prefer 60-cm Roux-en-Y gastro-jejunal anastomosis to total gastrectomy, because it is simpler and has less risks of malabsorption [7].

## 3 Conclusion

Gastro-colo-bronchial fistula is unreported after sleeve gastrectomy and the management is challenging, the surgeons may follow the same principles of management as in cases of gastrobronchial fistula, but we suggest more aggressive management and earlier surgical intervention together with the administration of stronger and broad spectrum antibiotics. Nutritional deficiencies must be corrected, and such patients must be treated with multidisciplinary team, with an extended duration of follow-up.

**Patient Perspective:** I had the worst chest infection in my life and sometimes I coughed up food particles, many surgeries were performed and I want to get back to my normal life again.

**Informed Consent:** Written informed consent was obtained from the patient for publication of this case report and accompanying images.

## Funding source

This research did not receive any funding from any resource.

## CRediT authorship contribution statement

**Ayad Ahmad Mohammed:** Data curation, Formal analysis, Writing - original draft. **Sardar Hassan Arif:** Data curation, Formal analysis, Writing - original draft.

## Declaration of competing interest

There is no conflict of interest.
